# Cell type- and tumor zone-specific expression of pVEGFR-1 and its ligands influence colon cancer metastasis

**DOI:** 10.1186/s12885-015-1130-3

**Published:** 2015-03-07

**Authors:** Caren Jayasinghe, Nektaria Simiantonaki, Charles James Kirkpatrick

**Affiliations:** 1 Institute of Pathology, University Medical Center, Johannes Gutenberg University, Langenbeckstr. 1, 55101 Mainz, Germany; 2 Department of Pathology, Laboratory Dr. Wisplinghoff, Geibelstr. 2, 50931 Cologne, Germany

**Keywords:** Colon cancer metastasis, VEGF, PlGF, VEGF-B, VEGFR-1, pVEGFR-1

## Abstract

**Background:**

Detailed knowledge of the essential pro-angiogenic biomolecules, the vascular endothelial growth factor (VEGF) family and its receptors, in the characteristically heterogeneous tumor tissue is a pre-requisite for an effective personalized target therapy. The effects of VEGF receptors after ligand binding are mediated through receptor tyrosine autophosphorylation. We determined the relevance of the VEGFR-1 activating pathway for colon cancer (CC) metastasis.

**Methods:**

The expression profiles of VEGFR-1, phosphorylated (activated) VEGFR-1 (pVEGFR-1^Tyr1048^, pVEGFR-1^Tyr1213^ and pVEGFR-1^Tyr1333^) and the VEGFR-1 ligands (VEGF, PlGF and VEGF-B) were investigated using immunohistochemistry in different tumor compartments (intratumoral - invasive front - extratumoral), cell types (tumor cells – macro- (large and small vessels) and the microvasculature (capillaries) - inflammatory cells) in human sporadic non-metastatic, lymphogenous metastatic and haematogenous metastatic CC.

**Results:**

VEGF and PlGF produced by tumor cells have an autocrine affinity for their receptor VEGFR-1. Subsequent PlGF-mediated receptor activation by autophosphorylation at Tyr1048 and Tyr1213 is a potential signaling pathway, which in turn seems to protect against distant metastasis and, in regions of tumor budding, additionally against lymph node metastasis. This autocrine link could be supported by possible formation of PlGF-VEGF heterodimers and PlGF-PlGF homodimers, which are known to have anti-metastatic properties. In contrast, in order to enhance their potential for distant metastasis tumor cells produce paracrine-acting VEGF-B. VEGFR-1 activation in tumor-associated macrovasculature but not capillaries appears to affect metastatic ability. Paracrine-mediated receptor autophosphorylation at Tyr1048 and Tyr1213 in small vessels located intratumorally and along the invasive front appears to be inversely correlated with metastasis, especially distant metastasis. Additionally, macrovessels are able to produce VEGFR-1 ligands, which influence the metastatic potential. Paracrine-acting VEGF-B production by intratumorally located small vessels and autocrine-acting PlGF production by extratumorally located small vessels seem to be associated with the non-metastatic phenotype. In contrast, VEGF-B-expressing extratumoral large and small vessels correlate with distant metastasis. Lymphocyte-associated VEGFR-1 expression in the invasive front without accompanying autophosphorylation could prevent against distant metastasis possibly by acting as a decoy and scavenger receptor.

**Conclusion:**

VEGFR-1 and its ligands participate in vascular, tumor cell-mediated and immuno-inflammatory processes in a complex biomolecule-dependent and tumor zone-specific manner and hence could influence metastatic behavior in CC.

## Background

Angiogenesis is a hallmark of cancer and is essential for tumor spread and life-threatening metastasis [1]. The major mediators of tumor angiogenesis are the vascular endothelial growth factor (VEGF) family and its receptors [2]. The use of VEGF pathway inhibitors to impair angiogenesis represents a clinically validated therapeutic strategy. However, such treatments are not completely curative, and a large number of tumors develop resistance or show recurrence after a progression-free period [3]. Contributory limiting factors for complete therapeutic success are the tumor heterogeneity and the complex cross-talk between tumor cells and the tumor microenvironment, which principally involves the tumor-associated vasculature and the peritumoral inflammatory reaction. A systematic analysis of the expression patterns of the ligands and receptors of the VEGF family in the tumor cells and the components of the tumor microenvironment *in situ* could contribute to a better understanding of the underlying interactive mechanisms determining tumor progressive behavior and subsequently help to improve the therapeutic approaches. In this context, the present study focusses on the expression profiles of members of the VEGF receptor-1 (VEGFR-1) activating pathway in colon cancer (CC) tissue.

VEGFR-1 is a member of the receptor tyrosine kinase (RTK) gene family and acts as a high affinity receptor for VEGF (often referred to as VEGF without a suffix), placenta growth factor (PlGF), and VEGF-B [4,5]. VEGFR-1 is composed of seven extracellular immunoglobulin homology domains, a single transmembrane region and an intracellular tyrosine kinase domain split by a kinase insert that is important for substrate recognition. It was originally identified by its important role in angiogenic processes. Further studies have demonstrated that the VEGFR-1 signaling pathway is also crucial for tumor growth, progression and metastasis. The mechanism by which the activation of VEGFR1 elicits these cellular events is not yet clearly understood. However, it is known that tyrosine autophosphorylation represents a crucial event in the activation of RTKs [6]. RTK activation is associated with ligand-mediated receptor dimerization, transphosphorylation and docking of signaling proteins to receptor phosphotyrosines. Residues of the C-terminal tail, including tyrosines (Tyr)1213 and 1333 and residues within the tyrosine kinase domain such as Tyr1048, have been identified as phosphorylation sites of VEGFR-1 [7,8]. Notably, in tumors there is also a possible oncogenic RTK activation by mutations and abnormally stimulated autocrine-paracrine loops [9]. These activation loops are stimulated when a RTK is abnormally expressed or overexpressed in the presence of its associated ligand or when there is an overexpression of the ligand in the presence of its cognate receptor. *In situ* data on the phosphorylated, activated status of VEGFR-1 in human tumor tissue are not available. Recently, specific antibodies for paraffin-embedded sections have been produced which detect endogenous levels of VEGFR-1 only when phosphorylated at the appropriate tyrosine residue. This offers the morphologist the possibility to localize those cells in a heterogeneous population which possess this functional phenotype.

The role of the most widely studied angiogenic factor, VEGF, in tumor angiogenesis via stimulation of VEGFRs expressed on tumor endothelium is well established [10,11]. VEGF stimulation activates endothelial proliferation, migration, survival and vascular permeability. Additionally, the hypothesis has been formulated that VEGF supports tumor growth and progression by acting directly through VEGFRs expressed on tumor cells. However, the significance of autocrine or paracrine acting VEGF in neoplastic tissue for tumor behavior is not fully elucidated.

PlGF is the second member of the VEGF family discovered and is highly expressed in the placenta throughout all stages of gestation [12,13]. PlGF binds exclusively to the VEGFR-1 with high affinity compared to VEGF and to VEGF-B. Moreover, if PlGF and VEGF are co-expressed in the same cell, they may generate PlGF/PlGF and VEGF/VEGF homodimers as well as PlGF/VEGF heterodimers. Each of these ligand pairs is able to bind and activate VEGFR-1, but receptor stimulation may lead to varying cellular responses. PlGF is produced by tumor cells, endothelial cells and other cells of the tumor stroma, including inflammatory cells. Although it is known that PlGF can stimulate tumor angiogenesis, until now the role of PlGF in tumor progression remains controversial.

VEGF-B, another ligand of VEGFR-1, seems to be a redundant and elusive member of the VEGF family [14]. Except for its ischemia-associated, myocardium-specific angiogenic activity, VEGF-B is minimally involved in angiogenesis in other organs. On the other hand, VEGF-B is a critical regulator of energy metabolism by regulating fatty acid uptake. Moreover, VEGF-B plays an important role in cell survival of vascular and non-vascular cells. Interestingly, VEGF-B is expressed in virtually all malignant tumor types, but its role in tumor biology appears limited [15].

In order to determine the relevance of the VEGFR-1 activating pathway for CC metastasis we investigated the expression profiles of the total and phosphorylated form of this receptor and its ligands in tumor cells, tumor-associated macro- (large and small vessels) and microvasculature (capillaries) and peritumoral inflammatory cells in 86 non-metastatic (N0/M0), lymphogenous (N+) and haematogenous (M+) metastatic, locally advanced CC. Taking tumor heterogeneity into consideration, the tumor tissue was subdivided in three separately investigated, strategically important compartments, namely tumor center (zone 1), invasive margin (zone 2) and tumor-free surrounding adipose cell-rich soft tissue (zone 3). Regarding the tumoral expression pattern we focused our attention on the topological staining distribution, especially on differences in staining intensity between the central tumor fraction and the invasive tumor margin. The expression patterns were assessed holistically in the light of previously published data about relevant features of CC such as tumor budding, tumor necrosis, peritumoral inflammation and vascular density [16].

## Methods

### Ethics statement

Ethical approval was granted by the Clinical Research Ethics Commitee of the federal state of Rhineland-Palatinate (Mainz, Germany). Written informed consent was obtained from all patients.

### Tissue samples

The CC tissue samples used in this study derived from 86 patients with an average age of 65.2 (range 52–83) undergoing elective surgery for sporadic (non-hereditary) CC at the University of Mainz during the years 1998–2003. Familial adenomatous polyposis (FAP), hereditary nonpolyposis colorectal cancer (HNPCC) and carcinomas associated with ulcerative colitis or Crohn’s Disease were exclusion criteria. All tumors were staged following the guidelines of the TNM Classification of Malignant Tumors. With respect to the T status all tumors investigated were T3 (infiltration of subserosa) and moderately differentiated (G2). According to metastatic status 37 of them were non-metastatic, 24 lymphogenous metastatic and 25 haematogenous metastatic CC at the time of diagnosis.

### Immunohistochemistry

All immunohistochemical reactions were conducted on formalin-fixed and paraffin-embedded samples.

*VEGF-B, PlGF and pVEGFR-1*^*Tyr1333*^*:* After deparaffination heat-induced epitope retrieval was performed in Tris-EDTA buffer pH 9,0 for 20 min. using a vegetable steamer. Non-specific binding was blocked by Dako REAL™ Peroxidase-Blocking Solution (Dako, Hamburg, Germany) prior to incubation with the primary antibody. For the immunohistochemical staining procedure DAKO REAL™EnVision™Detection System, Peroxidase/DAB+, Rabbit/Mouse was utilized following the manufacturer’s instructions. The primary antibodies, mouse monoclonal anti-VEGF-B (Santa Cruz Biotechnology, Inc., Santa Cruz, USA) and rabbit polyclonal anti-phosphoVEGFR-1 (pTyr1333; Abcam, Cambridge, UK) were applied at a dilution of 1:50 and 1:100 respectively for 1 h at room temperature. The primary antibody rabbit polyclonal anti-PlGF (Abcam) was applied at a dilution of 1:50 over night at 4°C.

*VEGF, VEGFR-1, pVEGFR-1*^*Tyr1048*^*and pVEGFR-1*^*Tyr1213*^: After deparaffination endogenous peroxidase activity was blocked with hydrogen peroxide. Heat-induced epitope retrieval was performed in citrate buffer pH 6,0 for 8 min. using a pressure cooker. The detection kits ZytoChem Plus HRP Kit, anti-Rabbit and ZytoChem Plus (HRP) Polymer Kit, anti-Mouse (Zytomed Systems, Berlin, Germany) were utilized following the manufacturer’s instructions. The primary antibodies were applied for 45 min. at room temperature and diluted as follows: mouse monoclonal anti-VEGF (Abcam) 1:40, rabbit monoclonal anti-VEGFR-1 (Y103, Abcam) 1:100, rabbit polyclonal anti-phosphoVEGFR-1 (pY1048, Abcam), 1:90 and rabbit polyclonal Anti-phosphoVEGFR-1 (pY1213, Ab-2, Merck, Darmstadt, Germany) 1:1000. Staining was completed with Novolink Max DAB (Polymer) Kit (Leica Biosystems, Wetzlar, Germany).

Sections were counterstained with Mayer's hematoxylin (Thermo Fisher Scientific, Fremont, USA). To prove the specificity of the immunoreactions, CC samples were stained solely with the secondary antibody, omitting the primary antibody, and these served as negative control.

Immunostaining reactions of each sample were evaluated independently by two authors (CJ and NS) without knowledge of the metastatic status. The endothelial and inflammatory cell staining was judged as either negative or positive. The intensity of the tumoral staining was scored on a semiquantitative scale from 0 to 2 depending on the investigated biomolecule (0: no staining, 1: weak staining, 2: strong staining). In most cases the staining was homogeneous. In those cases where heterogeneous staining was observed, that level of staining intensity which was visible in more than 50% of the cells was chosen for the classification into a defined group. In those cases (<5%) in which the evaluation results of the two independent authors (CJ and NS) were different, the specimens were re-evaluated together and a consistent score was found.

### Histopathological analysis

*Tumor budding* was defined as disseminated single tumor cells and oligocellular tumor clusters (≤5 tumor cells) at the invasive margin.

*Capillaries (microvessels)* were vessels with clearly defined lumina or linear vessel shape lacking a definable smooth muscle wall.

*Small vessels (macrovessels)* were vessels with narrow lumina and up to five well definable smooth muscle layers.

*Large vessels (macrovessels)* were arteries with a thick muscular wall.

#### Statistical analysis

Statistical significance was assessed using Fisher's exact test. p < 0.05 was considered to be statistically significant. The correlations between expression of VEGFR-1 and ligands were assessed with the Spearman’s rank test.

## Results

### Tumor cell- associated VEGFR-1 activation in CC tissue

The VEGFR-1 ligands VEGF, PlGF and VEGF-B were expressed in the cytoplasm of tumor cells by 82%, 83% and 26% of the CC, respectively (Table 1). In most of the tumors VEGF was detected with uniform staining intensity and distribution within the three comparative tumor fractions. PlGF overexpression and VEGF-B absence each correlated significantly with non-metastatic status in comparison to distant metastatic spread (p = 0.04 and p = 0.02, respectively). However, it is worth mentioning that the percentage distribution between negative and positive PlGF expression was approximately of the same order in the non-metastatic and metastatic groups (no statistical significance). Correlation analysis displayed existing moderate VEGF/VEGFR-1 and weak PlGF/VEGFR-1 ligand-receptor affinity (r = 0.5, p = 0.0001 as well as r = 0.3, p = 0.007 in tumor center and r = 0.3 and p = 0.02 in tumor budding, respectively; Table 2). The documented PlGF/VEGFR-1 affinity was observed exclusively in the metastatic cases. It is known, that if PlGF and VEGF are co-expressed in the same cell, they may generate PlGF/PlGF and VEGF/VEGF homodimers as well as PlGF/VEGF heterodimers [13]. Each of these ligand formations is able to bind and activate VEGFR-1 but receptor stimulation may lead to different cellular responses. The percentage distribution of PlGF/VEGF dimers within the various CC groups was approximately the same, without statistical significance (Table 3).

**Table 1:** **Percentage distribution of the VEGFR-1 ligands in tumor cells of CC tissue**

	Score	N0/M0 (%)	N+ (%)	N0/M0 vs. N + p	M+ (%)	N0/M0 vs. M + p	CC (%)
**Tumor center**							
**VEGF**	0	14	25	NS 0.3	16	NS 1.0	18
	1	68	54		60		82
	2	18	21		24		
**PlGF**	0	14	21	NS 0.5	16	*0.04*	17
	1	48	54		72		83
	2	38	25		12		
**VEGF-B**	0	84	83	NS 1.0	56	*0.02*	74
	1	16	17		44		26
	2	0	0		0		
**Tumor budding**							
**VEGF**	0	14	15	NS 1.0	17	NS 1.0	15
	1	72	60		58		85
	2	14	25		25		
**PlGF**	0	11	18	NS 1.0	16	*0.03*	15
	1	50	46		72		85
	2	39	36		12		
**VEGF-B**	0	75	80	NS 1.0	56	NS 0.06	70
	1	14	15		36		30
	2	11	5		8		

The intensity of the tumoral staining was scored on a semiquantitative scale from 0 to 2 for the investigated biomolecule (0: no staining, 1: weak staining, 2: strong staining). For the statistical analysis using Fisher’s exact test the examined cases were separated into two groups characterized by a negative/positive expression for VEGF and VEGF-B or negative, low/high expression for PlGF. The line in the score (staining intensity) column indicates this dichotomization for each biomolecule. The line in the column “CC %” indicates the percentage distribution of colon carcinomas with negative and positive expression of each biomolecule. p < 0.05 was taken as statistically significant. NS, not significant.

**Table 2:** **Numerical distribution of ligand/VEGFR-1 correlations in tumor cells of CC tissue**

	VEGFR-1	VEGF		p	PlGF		p	VEGF-B		p
		+	-	(r)	+	-	(r)	+	-	(r)
**Tumor center**										
**CC**	+	68	8	*0.0001*	66	10	*0.007*	23	56	NS 0.4
	-	3	7	(0.5)	4	5	*(0.3)*	1	6	(0.1)
**N0/M0**	+	32	4	*0.009*	31	5	NS 0.7	6	30	NS 0.7
	-	0	1	*(0.4)*	1	0	(0.07)	0	1	(0.07)
**N+**	+	16	3	*0.04*	17	3	*0.009*	7	15	NS 0.4
	-	2	3	*(0.4)*	1	3	*(0.5)*	0	2	(0.1)
**M+**	+	20	1	*0.0001*	18	2	*0.04*	10	11	NS 0.6
	-	1	3	*(0.7)*	2	2	*(0.4)*	1	3	(0.1)
**Tumor budding**										
**CC**	+	59	10	NS 0.4	60	7	*0.02*	21	47	NS 0.6
	-	2	1	(0.01)	2	3	*(0. 4)*	1	4	(0.05)
**N0/M0**	+	24	3	*0.01*	24	3	NS 0.7	7	20	NS 0.6
	-	0	1	*(0.5)*	1	0	(0.09)	0	1	(0.1)
**N+**	+	16	3	NS 0.7	17	2	*0.01*	4	15	NS 0.6
	-	1	0	(0.1)	0	1	(0.5)	0	1	(0.1)
**M+**	+	19	4	NS 0.7	19	2	*0.01*	10	12	NS 0.7
	-	1	0	(0.1)	1	2	*(0.5)*	1	2	(0.07)

Positive tumoral expression of VEGFR-1 is positively correlated with positive tumoral VEGF expression in the tumor center and positive tumoral PlGF expression in the tumor center and tumor budding regions. r = Spearman’s rank correlation coefficient. p < 0.05 was taken as statistically significant. NS, not significant.

**Table 3:** **Percentage distribution of potential autocrine PlGF/VEGF dimer formation in tumor cells of CC tissue**

	N0/M0 (%)	N+ (%)	p	M+ (%)	p
**PlGF/VEGF**	75	63	NS 0.4	74	NS 0.8
**PlGF/PlGF**	14	25	NS 0.3	13	NS 1.0
**VEGF/VEGF**	11	12	NS 1.0	13	NS 1.0

In a next step we investigated the VEGFR-1 ligand expression profiles in the tumor budding regions, which reflect the spreading capacity of tumor cells. Here, the percentage distribution of cases with positive VEGFR-1 ligand immunoreactivity was similar to the tumor center, namely 85% for VEGF and PlGF and 30% for VEGF-B (Table 1). Consequently, the correlations among the metastatic categories remained constant, except for VEGF-B with a difference in the expression pattern between N0/M0 and M+ CC just below the level of statistical significance (p = 0.06).

**Figure 1:**
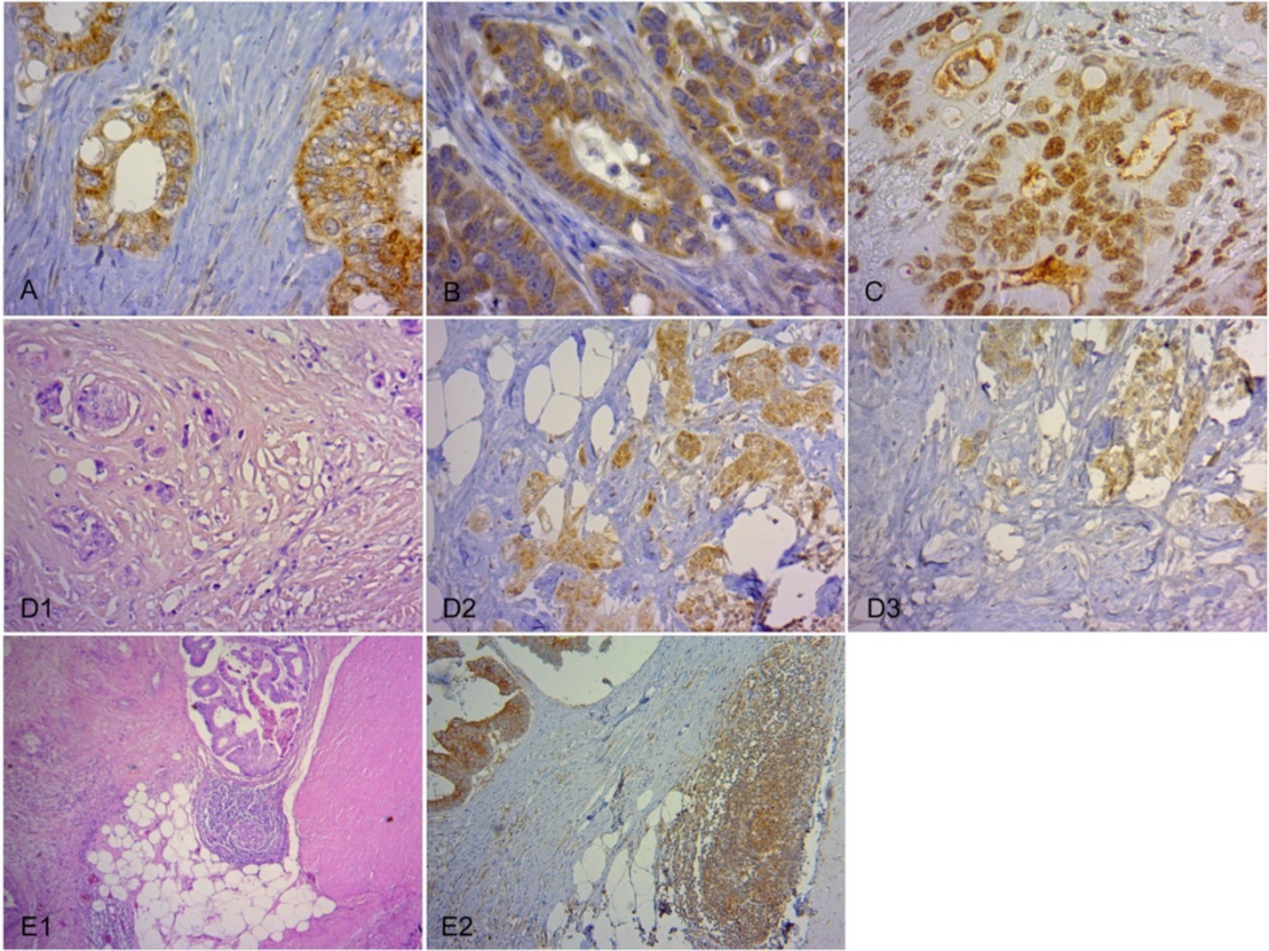
**Immunohistochemical staining of pVEGFR-1 in tumor cells and VEGFR-1 in inflammatory cells of CC tissue. (A)** Characteristic pVEGFR-1^Tyr1048^ expression in tumor cells with membranous and cytoplasmic immunostaining (x 400). **(B)** Characteristic pVEGFR-1^Tyr1213^ expression in tumor cells with membranous and cytoplasmic immunostaining (x 400). **(C)** Characteristic pVEGFR-1^Tyr1333^ expression in tumor cells with nuclear immunostaining (x 400). **(D)** pVEGFR-1 expression in tumor cells in tumor budding regions. Tumor budding was defined as single tumor cells and oligocellular tumor cell clusters along the invasive margin (D1, H.E., x 200). Expression of pVEGFR-1^Tyr1048^ (D2, x 200) and pVEGFR-1^Tyr1213^ (D3, x 200) in tumor budding regions. **(E)** Characteristic VEGFR-1 expression in inflammatory cells. Lymph follicles along the invasive front (E1, H.E., x 40) with VEGFR-1 immunopositivity (E2, x 100) in a non-metastatic CC case.

In the tumor center and tumor budding regions 87% and 94% of the CC, respectively, have shown a positive VEGFR-1 cytoplasmatic expression (Table 4). Negative VEGFR-1 expression in the tumor core was associated with lymphogenous metastasis (p = 0.03). From the 37 investigated N0/M0 cases 27 CC exhibited tumor budding. Interestingly, from the 10 cases without this histopathological feature, 9 tumors were characterized by positive VEGFR-1 expression. Consequently, in the tumor budding regions significant differences did not exist between non-metastatic and metastatic status. The VEGFR-1 phosphorylated at Tyr1048 and Ty1213 exhibited a submembranous accentuated cytoplasmatic and at Tyr1333 a specific nuclear immunoreactivity (Figure1A-C). Positive pVEGFR-1^Tyr1048^, pVEGFR-1^Tyr1213^ and pVEGFR-1^Tyr1333^ expression was seen in 74%, 64% and 55%, respectively (Table 4). Negative pVEGFR-1^Tyr1048^ and pVEGFR-1^Tyr1213^ immunoreactivity was significantly correlated with distant metastatic stage (p = 0.01). In the tumor budding regions the percentage distribution of positive pVEGFR-1 expression in the same sequence as above was 71%, 64% and 47%, respectively, and thus almost identical (Table 4, Figure 1D). From the 4 N+ CC without the presence of tumor budding 3 expressed strong immunostaining levels. This led to an additional statistical significance for pVEGFR-1^Tyr1048^ in tumor budding regions between N0/M0 and N+ CC (p = 0.01). pVEGFR-1^Tyr1333^ immunoreactivity had similar immunointensity distribution throughout all comparative groups.

**Table 4:** **Percentage distribution of VEGFR-1 and pVEGFR-1 in tumor cells of CC tissue**

	Score	N0/M0 (%)	N+ (%)	N0/M0 vs. N + p	M+ (%)	N0/M0 vs. M + p	CC (%)
**Tumor center**							
**VEGFR-1**	0	3	21	*0.03*	16	NS 0.1	13
	1	11	21		12		87
	2	86	58		72		
**pVEGFR-1** ^**Tyr1048**^	0	11	29	NS 0.09	30	*0.01*	26
							74
	1	59	54		44		
	2	30	17		16		
**pVEGFR-1** ^**Tyr1213**^	0	19	38	NS 0.1	52	*0.01*	36
							64
	1	64	46		32		
	2	17	16		16		
**pVEGFR-1** ^**Tyr1333**^	0	38	46	NS 0.8	52	NS 0.3	45
							55
	1	49	33		44		
	2	13	21		4		
**Tumor budding**							
**VEGFR-1**	0	0	5	NS 0.4	12	NS 0.1	6
							94
	1	15	15		12		
	2	85	80		76		
**pVEGFR-1** ^**Tyr1048**^	0	4	30	*0.01*	40	*0.003*	29
							71
	1	66	50		44		
	2	30	20		16		
**pVEGFR-1** ^**Tyr1213**^	0	19	38	NS 0.2	52	*0.01*	36
							64
	1	64	46		32		
	2	17	16		16		
**pVEGFR-1** ^**Tyr1333**^	0	46	50	NS 1.0	64	NS 0.3	53
							47
	1	43	25		32		
	2	11	25		4		

The intensity of the tumoral staining was scored on a semiquantitative scale from 0 to 2 for the investigated biomolecule (0: no staining, 1: weak staining, 2: strong staining). For the statistical analysis using Fisher´s exact test the examined cases were separated into two groups characterized by a negative/positive expression. The line in the staining intensity column indicates this dichotomization for each biomolecule. The line in the column “CC %” indicates the percentage distribution of colon carcinomas with negative and positive expression of each biomolecule. p < 0.05 was taken as statistically significant. NS, not significant.

Since a concomitant VEGFR-1/pVEGFR-1 immunopositivity can be interpreted as a potentially ligand-dependent tyrosine autophosphorylation, co-expression profiles were also analyzed. These analyses revealed the same significant correlations as described above, but with an additional significance concerning the association between negative VEGFR-1/pVEGFR-1^Tyr1213^ and N+ CC in the presence of tumor budding (p = 0.02, Table 5).

**Table 5:** **Numerical and percentage distribution of VEGFR-1/pVEGFR-1 co-expression tumor cells**

	N0/M0 n (%)	N+ n (%)	N0/M0 vs. N + p	M+ n (%)	N0/M0 vs. M + p
**Co-expression in the tumor center**					
VEGFR-1+/pVEGFR-1^Tyr1048^+	36/32 (89)	19/13 (68)	NS 0.08	21/13 (62)	*0.02*
VEGFR-1+/pVEGFR-1^Tyr1213^+	36/28 (78)	19/12 (63)	NS 0.3	21/10 (48)	*0.02*
VEGFR-1+/pVEGFR-1^Tyr1333^+	36/22 (61)	19/9 (47)	NS 0.4	21/10 (48)	NS 0.4
**Co-expression in tumor budding regions**					
VEGFR-1+/pVEGFR-1^Tyr1048^ +	26/25 (96)	19/13 (68)	*0.02*	22/14 (64)	*0.005*
VEGFR-1+/pVEGFR-1^Tyr1213^ +	26/21 (81)	19/9 (47)	*0.02*	22/11 (50)	*0.01*
VEGFR-1+/pVEGFR-1^Tyr1333^ +	26/13 (50)	19/9 (47)	NS 1.0	22/9 (41)	NS 0.6

n: total number of VEGFR-1 positive cases/total number of pVEGFR-1 positive cases with concomitant VEGFR-1 positivity. p < 0.05 statistically significant, NS not significant.

### Inflammatory cell-associated VEGFR-1 activation in CC tissue

Of the three VEGFR-1 ligands only PlGF was markedly expressed on inflammatory cells – independent of the tumor zone – on average in 80% of the cases (Table 6). VEGF expression was sporadic and occurred in less than 10% of all cases. None of the CC showed VEGF-B immunopositivity. VEGFR-1 and pVEGFR-1 revealed immunoreactivity in different frequencies, ranging from 33% to 83% intratumorally and from 50% to 95% along the invasive front. The only significant difference was observed in the tumor border, where in 92% of the non-metastatic CC inflammatory cells were VEGFR-1 positive whereas only 68% of the cases with distant metastasis had a positive immunoreaction (p = 0.02, Figure 1E). Based on correlation analysis no significance between PlGF and VEGFR-1 could be demonstrated (data not shown).

**Table 6:** **Percentage distribution of the VEGFR-1 ligands, VEGFR-1 and pVEGFR-1 in inflammatory cells of CC tissue**

	N0/M0 (%)		N+ (%)				M+ (%)			
	z1	z2	z1	N0/M0 vs. N + p	z2	N0/M0 vs. N + p	z1	N0/M0 vs. M + p	z2	N0/M0 vs. M + p
**VEGF**	3	5	5	NS 1.0	18	NS 0.2	5	NS 1.0	8	NS 1.0
**PlGF**	69	84	78	NS 0.7	95	NS 0.2	71	NS 1.0	79	NS 0.7
**VEGF-B**	0	0	0		0		0		0	
**VEGFR-1**	67	92	50	NS 0.2	82	NS 0.4	59	NS 0.6	68	*0.02*
**pVEGFR-1** ^**Tyr1048**^	83	95	68	NS 0.3	91	NS 0.6	68	NS 0.2	80	NS 0.1
**pVEGFR-1** ^**Tyr1213**^	60	64	33	NS 0.1	50	NS 1.0	50	NS 0.6	56	NS 0.8
**pVEGFR-1** ^**Tyr1333**^	75	86	67	NS 0.5	68	NS 0.1	72	NS 1.0	68	NS 0.1

z1 = zone 1, z2 = zone 2. p < 0.05 statistically significant. NS, not significant.

### Vasculature-associated VEGFR-1 activation in CC tissue

The vascular expression profiles of the VEGFR-1 activating pathway were investigated separately in the three vessel types (large vessels, small vessels and capillaries) within the three zones.

**Figure 2:**
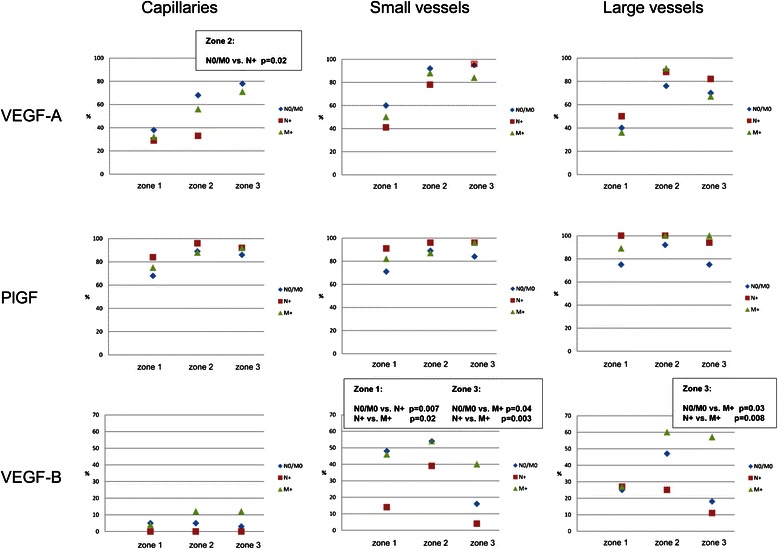
**Graphical presentation of percentage distribution of the VEGFR-1 ligands in the vasculature of CC tissue.**

**Figure 3:**
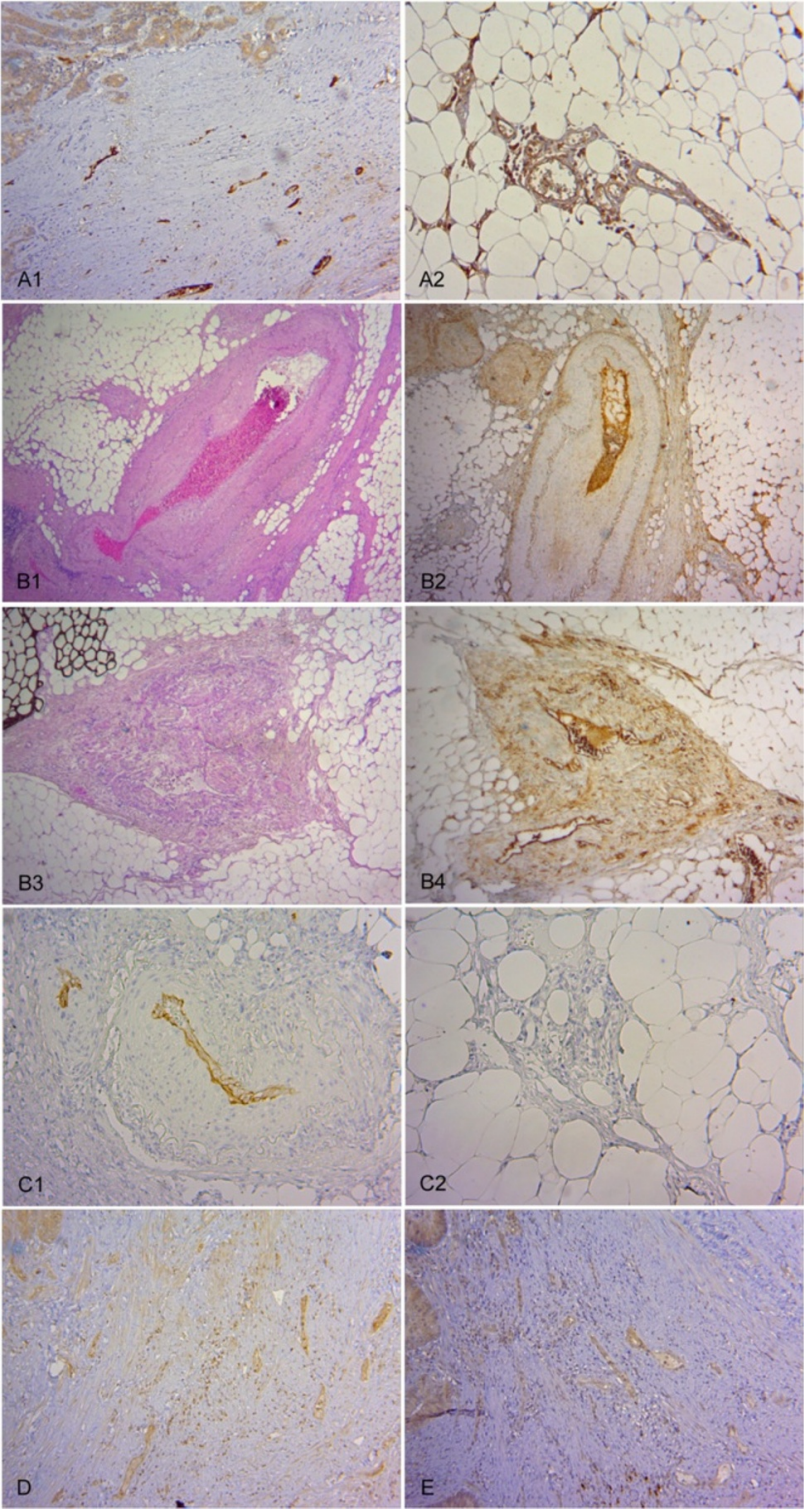
**Immunohistochemical staining of the VEGFR-1 ligands and pVEGFR-1 in the vasculature of CC tissue. (A)** Characteristic endothelial VEGF expression. VEGF positive intratumoral microvascular vessels with predominantly compressed lumina (A1, x 100) and extratumoral microvascular vessels with open lumina (A2, x 100). **(B)** Characteristic endothelial PlGF expression: Macrovascular vessels with arteriosclerotic changes (B1, H.E., x 40) and PlGF immunopositivity (B2, x 40) as well as altered macrovascular vessels with discontinuous, hypoplastic smooth muscle cell layer (B3, H.E., x 40) and PlGF immunopositivity (B4, x 40). **(C)** Characteristic endothelial VEGF-B expression: Small and large vessels with VEGF-B immunopositivity (C1, x 100) and capillaries with absent VEGF-B expression (C2, x100). **(D)** Characteristic endothelial pVEGFR-1^Tyr1048^ expression in small intratumoral vessels (x 100). **(E)** Characteristic endothelial pVEGFR-1^Tyr1213^ expression in small intratumoral vessels (x 100).

Concerning VEGF, there were markedly more cases with VEGF-expressing macro- and microvascular vessels (N0/M0, M+) at the invasive front compared to the tumor center (Figure 2). In nodal-positive CC (N+) this expression was observed only in the macrovasculature. In comparison with lymph node metastatic CC almost twice as many non-metastatic carcinomas displayed positive VEGF staining of the microvascular vessels in zone 2 (p = 0.02). Intratumoral capillaries within the desmoplastic stroma showed predominantly compressed lumina, although some were partly open (Figure 3A1,2). In contrast, a clear dominance of capillaries with open lumina could be seen in zone 3.

In more than two-thirds of the cases a positive endothelial PlGF reaction was seen in both large and small vessels, as well as in capillaries (Figure 2). However, no significant differences could be established with respect to the metastatic status. In addition to PlGF-positive atherosclerotic large vessels, altered blood vessels with hypoplastic and disorganized muscle wall layers were also present (Figure 3B1-4). These immature vessels were in almost all cases PlGF-positive.

Staining of VEGF-B was seen in the macrovasculature, but, with exception of single tumor cases, not in the microvasculature (Figures 2 and 3C1,2). Making a comparison between cases without metastases (N0/M0) and distant metastases (M+) on the one hand and cases with lymph node metastases (N+) on the other hand showed two distinguishing features. In nodal metastatic CC there were significantly less cases with VEGF-B positive small vessels in the tumor center (p = 0.007 for N0/M0 vs. N+ and p = 0.02 for N+ vs. M+). In the distant metastasizing CC significantly more cases revealed VEGF-B-positive small vessels (p = 0.04 for N0/M0 vs. M+ and p = 0.003 for N+ vs. M+) and large vessels (p = 0.03 for N0/M0 vs. M+ and p = 0.008 for N+ vs. M+) in the extratumoral adipose tissue (Figure 2).

**Figure 4:**
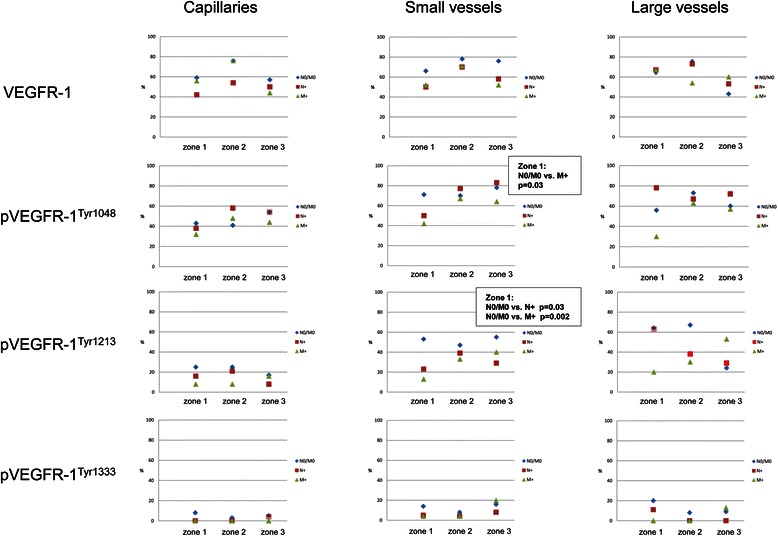
**Graphical presentation of percentage distribution of VEGFR-1 and pVEGFR-1 in the vasculature of CC tissue.**

Positive vascular VEGFR-1 immunoreactivity in all segments of the vascular network was observed with a moderate increase of cases with VEGFR-1-positive capillaries and small vessels from zone 1 to zone 2 (Figure 4). No significant correlation between non-metastatic and metastatic status was noted.

The three zones exhibited endothelial expression of pVEGFR-1^Tyr1048^ in all segments of the vascular system (Figures 3D, 4). In comparison with N0/M0 carcinomas only a small number of M + −cases presented phosphorylated receptor-positive small vessels in the tumor center (p = 0.03).

Endothelial expression of pVEGFR-1^Tyr1213^ was detectable in the macrovasculature in all three zones (Figures 3E and 4). Phosphorylated receptor-positive small vessels in the tumor center were significantly more often detected in non-metastatic cases (p = 0.03 N0/M0 vs. N+ and p = 0.002 N0/M0 vs. M+). Positive capillary pVEGFR-1^Tyr1213^ immunoreactivity was present only in a small number of cases.

Endothelial expression of pVEGFR-1^Tyr1333^ was observed very infrequently in all vascular segments (Figure 4).

Vascular ligand/VEGFR-1 correlation analysis revealed that in zone 3 located PlGF-expressing capillaries and small vessels were significantly correlated with their receptor expression (r = 0.4, p = 0.0008 and r = 0.3, p = 0.01, respectively, data not shown). PlGF-VEGFR-1 co-expression in extratumoral small vessels was seen more frequently in non-metastatic cases (84% in N0/M0 versus 61% in N+ (p = 0.07) and 57% for M+ (p = 0.03)).

VEGFR-1/pVEGFR-1 co-expression analysis showed statistical significance for small vessels but not for capillaries (Table 7). Small intratumoral vessels with combined VEGFR-1/pVEGFR-1^Tyr1048^ expression occurred significantly more often in non-metastatic CC compared with lymphogenous and haematogenous metastatic cases (p = 0.01 and p = 0.02, respectively). Furthermore, metastasis-free CC revealed in a larger number of cases a simultaneous positive VEGFR-1/pVEGFR-1^Tyr1213^ immunoreaction in small vessels in the tumor center and along the invasive margin compared to distant-metastatic cases (p = 0.05 and p = 0.04, respectively). As there was only a small number of cases with co-expression in large vessels, this vessel type was not considered for statistical evaluation.

**Table 7:** **Numerical and percentage distribution of VEGFR-1/pVEGFR-1 co-expression in the vasculature**

		N0/M0 n (%)	N+ n (%)	N0/M0 vs. N + p	M+ n (%)	N0/M0 vs. M + p
**VEGFR1+/pVEGFR-1** ^**Tyr1048**^ **+**						
Capillaries	zone 1	22/9 (41)	10/4 (40)	NS 1.0	14/7 (50)	NS 0.7
	zone 2	28/11 (39)	13/8 (62)	NS 0.3	19/13 (68)	NS 0.08
	zone 3	21/12 (57)	12/8 (67)	NS 0.7	11/5 (45)	NS 0.7
small vessels	zone 1	23/19 (83)	11/4 (36)	*0.01*	12/5 (42)	*0.02*
	zone 2	29/20 (69)	15/12 (80)	NS 0.5	17/14 (82)	NS 0.5
	zone 3	27/22 (81)	14/13 (93)	NS 0.6	13/7 (54)	NS 0.2
**VEGFR-1+/pVEGFR-1** ^**Tyr1213**^ **+**						
Capillaries	zone 1	21/6 (28)	8/2 (25)	NS 1.0	14/2 (14)	NS 0.4
	zone 2	27/7 (26)	12/3 (25)	NS 1.0	18/1 (6)	NS 0.1
	zone 3	27/16 (59)	14/5 (36)	NS 0.2	13/7 (54)	NS 1.0
small vessels	zone 1	22/12 (55)	11/3 (27)	NS 0.3	11/2 (18)	*0.05*
	zone 2	28/13 (46)	16/8 (50)	NS 1.0	16/2 (13)	*0.04*
	zone 3	27/16 (59)	14/5 (36)	NS 0.2	13/7 (54)	NS 1.0

n: total number of VEGFR-1 positive cases/total number of pVEGFR-1 positive cases with concomitant VEGFR-1 positivity. p < 0.05 statistically significant, NS not significant.

**Figure 5:**
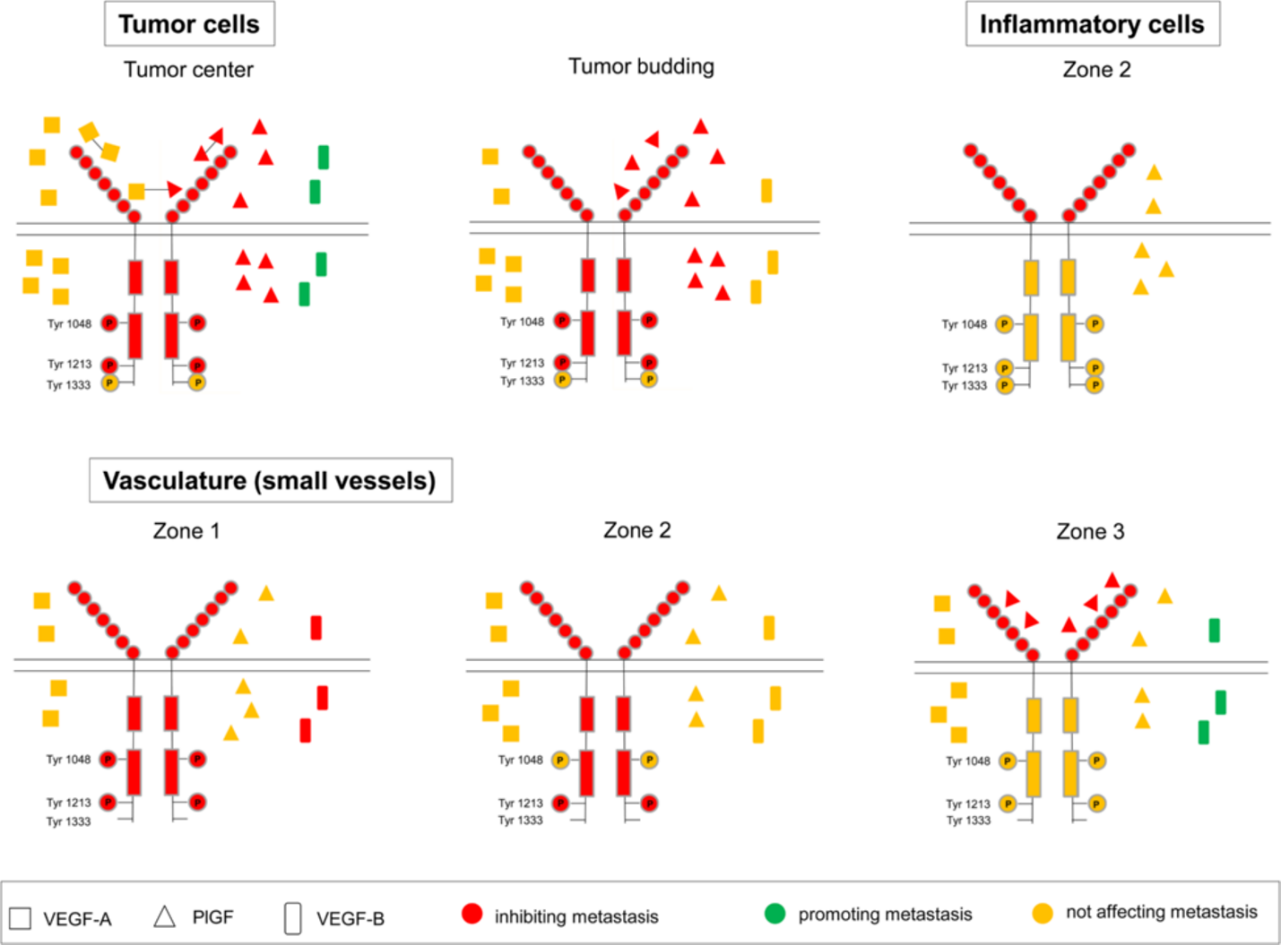
**Schematic presentation of VEGFR-1 activation in CC tissue and its association with metastasis.** VEGF produced in the tumor center and PlGF produced intratumorally and in tumor budding regions by tumor cells have an autocrine affinity for their receptor VEGFR-1. Subsequent PlGF-mediated receptor activation by autophosphorylation at Tyr1048 and Tyr1213 is a potential signaling pathway, which in turn seems to inhibit distant metastasis and, in regions of tumor budding, additionally lymph node metastasis. This autocrine link could be supported by possible formation of PlGF-VEGF heterodimers and PlGF-PlGF homodimers, which are known to have anti-metastatic properties. In contrast, in order to enhance their potential for distant metastasis, tumor cells in the tumor center produce paracrine-acting VEGF-B. Inflammatory cell associated VEGFR-1 expression in the invasive front (zone 2) without accompanying autophosphorylation could inhibit distant metastasis possibly by acting as a decoy and scavenger receptor. In small vessels located intratumorally (zone 1) paracrine-mediated receptor autophosphorylation at Tyr1048 and Tyr1213 and paracrine-acting VEGF-B production appear to be associated with a non-metastatic phenotype. In small vessels located along the invasive front (zone 2) paracrine-mediated receptor autophosphorylation at Tyr1213 may cause inhibition of metastasis. Autocrine-acting PlGF production by small vessels located extratumorally (zone 3) appears to be associated with a non-metastatic phenotype. In contrast, VEGF-B-expressing extratumoral small vessels correlate with distant metastasis.

Figure 5 represents a summary of our results in schematic form.

## Discussion

This study investigated the tumor cell-, inflammatory cell- and vasculature-associated expression of total and phosphorylated VEGFR-1 and its ligands in different compartments of colon cancer tissue in relation to the metastatic status. The accentuated macrovascular VEGF-expression in the extratumoral tissue emphasizes the important role of the tumor-surrounding area for VEGF-controlled blood vessel-related processes, which are crucial to provide the tumor with an adequate supply of oxygen and nutrients. Additionally, the large number of microvascular VEGF-expressing cases in the extratumoral region in nodal metastatic CC underlines the relevance of VEGF-controlled extratumoral microvasculature for lymph node metastasis. Microvessels in the immediate tumor vicinity with their open lumina are probably the most favorable site of entry and further transport of tumor cells compared to the mostly collapsed intratumoral microvessels, reflecting a mechanical stress situation of the muscle-layer free vasculature in desmoplastic tumor tissue. This topological peculiarity of the microvasculature in CC was clearly documented in the histological examination of the tumor tissue. Tumor cells also expressed VEGF in more than 75% of the CC, but without association with the metastatic status. Lack of significant correlation between endothelial as well as epithelial VEGF expression and CC metastasis in our study is in accordance with the results of several research groups [17-20]. There are, however, other reports describing a significant correlation between VEGF expression and lymph node as well as distant metastasis in CC [21,22]. In most publications, a detailed study of the cell type-related VEGF-expression was omitted. In our opinion, precise evaluation and characterization of the cell subtypes within the tumor tissue showing VEGF-immunopositivity could contribute to a better understanding of the paracrine and autocrine functions of this factor for tumor progression. Although the most potent angiogenic factor, VEGF expression in microvascular endothelial cells was not associated with metastatic status in our study, thus supporting previously published data showing no correlation between microvascular density (MVD) and metastatic stage in CC [16]. Since MVD analysis is the morphological gold standard to assess angiogenesis in human tumors, these results clearly indicate that angiogenesis alone is not able to promote metastasis in CC.

Recently, it has been shown that VEGF also exhibits immunosuppressive properties by inducing the accumulation of immature dendritic cells, myeloid-derived suppressor cells, regulatory T cells and inhibition of T lymphocyte migration to the tumor [23]. Data on VEGF involvement in immuno-inflammatory responses in colon tumor tissue are very rare. In one study VEGF-overexpression was observed in lymphocytes along the invasive tumor front of CC [18]. We found only a sporadic, non-specifically located inflammatory cell-associated VEGF expression in the investigated CC cases. Further studies with regard to the VEGF isoforms are required to verify the immunomodulatory properties of this factor. VEGF-B was not involved in the peritumoral inflammatory response. In contrast, an abundant PlGF-expression of inflammatory cells in the tumor center and especially the marginal tumor portion was demonstrated without effects on metastatic behavior. VEGFR-1 expression in the invasive front, especially in the non-metastatic cases showed significant differences in comparison to the distant-metastatic CC. Since a significant ligand/receptor correlation was lacking, an autocrine PlGF/VEGFR-1 link as an appropriate metastasis-limiting tool can be excluded. Likewise, pVEGFR-1 expression was not associated with metastatic spread. Taking the results together, lymphocyte-associated VEGFR-1 expression at the tumor-host interface in almost all non-metastatic CC underlines the possible importance of this receptor for preventing distant metastasis. Apart from conceivably underlying immunomodulatory mechanisms, a function as decoy and scavenger receptor for pro-metastatic acting VEGFR-1 ligands from the tissue vicinity might also be possible. In accordance with our results, there are several reports indicating that VEGFR-1 is expressed on different T cell subsets, suggesting its potential importance in immunity [24,25].

In a conspicuously large number of non-metastatic and metastatic cases all tumor zones displayed PlGF-positive micro- and macrovasculature. In addition to its already well documented angiogenic properties, these results provide evidence for an involvement of PlGF in the tumor vascularization process. In this context, in infarcted myocardial tissue sufficient endothelial PlGF production of autochthonous vessels within the necrotic myocardium was associated with an improvement in cardiac function [26]. In extratumoral small vessels a close correlation between VEGFR-1 and PlGF was documented, with significantly more non-metastatic than metastatic cases revealing PlGF/VEGFR-1 co-expression. Thus, an active autocrine PlGF/VEGFR-1 link in the macrovasculature adjacent to the tumor seems to exist in CC, which could protect against metastasis. It is known that PlGF also has arteriogenic properties by inducing the formation of large, stable blood vessels and medium-size collaterals after ischemia [27,28]. Whether the PlGF expression in the large and small vessels in our study represents an arteriogenic potential of this factor can only be speculated on at present. In one of our previously reported studies on the same cohort a third of the investigated CC showed altered vessels with a discontinuous, hypoplastic muscle wall layer, which could reflect immature tumor vascular entities possibly in the course of arteriogenesis [16]. Interestingly, almost all of them were PlGF-positive.

Recently, we reported that VEGF-B might also be an important factor in ensuring a functional blood supply for tumor survival in the absence of capillary participation [29]. In the present study these observations could be confirmed, since there was only a macrovascular but no capillary VEGF-B-expression. It is known that VEGF-B promotes fatty acid transport across the endothelium and lipid transport into tissues with an elevated rate of cellular metabolism [14]. In our study the autochthonous large and small vessels of the extratumoral tissue expressed VEGF-B significantly more frequently in distant-metastatic carcinomas. A possible explanation for this finding could be an increased fatty acid transportation from the subserosal adipose tissue to the tumor tissue, which had typically a low intratumoral small vessel density and abundant tumor necroses [16]. In the lymphogenous-metastatic carcinomas intratumoral VEGF-B-positive small vessels were present in a significantly reduced number of cases. This tumor tissue was previously characterized by a high extratumoral large vessel density and relatively sparse tumor necrosis. It is probable that the tumor center in these carcinomas has a sufficient blood supply and consequently requires reduced lipid uptake. We suppose that VEGF-B could fulfil a balancing regulatory function in lipid transport between energy-consuming and energy-providing segments of the colonic tumor tissue.

In our study, PlGF was significantly overexpressed in tumor cells of non-metastatic tumors in comparison with distant metastatic cases. Escudero-Esparza et al. reported similar results in CC at RNA-level with high expression in the earliest stages and remarkably low levels in the presence of distant metastases [30]. These findings point to a possible preventive role of PlGF secreted by the tumor cells themselves in CC. In this context, PlGF-overexpressing human colon tumor cells growing orthotopically in mice, inhibited angiogenesis, growth and metastasis by an increase of PlGF homodimers and PlGF/VEGF heterodimers [31]. In another experimental study, tumor cells expressing the heterodimeric form of PlGF/VEGF were found to be functionally inactive and lacked the ability to induce angiogenesis *in vitro* and *in vivo* [32]. It has also been reported that synthesis of both factors VEGF and PlGF in the same cell may generate PlGF/VEGF heterodimer forms [13]. In the present study autocrine formation of PlGF/PlGF homodimers and PlGF/VEGF heterodimers by tumor cells was about 88% in each comparative group. Concurrently, tumoral VEGFR-1 and pVEGFR-1^Tyr1048^ as well as pVEGFR-1^Tyr1213^ expression and co-expression was significantly correlated with distant metastasis. Thus, the detected metastasis-preventing role of PlGF could at least in part be due to receptor activation by PlGF dimers, having a negative effect on distant organ spread. However, since other *in situ* and experimental studies found a PlGF-stimulated, enhanced metastatic phenotype in cancer cells, additional analyses are clearly needed for a further understanding of the complex role of tumoral PlGF-expression [33-36].

VEGF-B positive cancer cells were detected only in 25% of the tumors, but significantly more frequently in cases with distant metastasis. *In vitro* studies demonstrated that VEGF-B led to significant induction of cell motility and invasiveness of colon carcinoma cell lines and epithelial to mesenchymal transition (EMT) in pancreatic carcinoma cell lines [37,38]. Tumor budding is thought to reflect the biological process of EMT as a manifestation of increased invasiveness [39]. Notably, the presently documented VEGF-B expression in distant metastatic tumors was associated in about two thirds of the cases with high tumor budding, while almost all VEGF-B positive N0/M0- and N + −CC displayed low tumor budding. Additionally, in the tumor budding regions the correlation between VEGF-B immunopositivity and distant metastasis was just below statistical significance. These observations suggest a synergistic auxiliary effect of VEGF-B in conjunction with high tumor budding for processes promoting distant CC metastasis.

The effects of VEGF are partly, and in the case of PlGF and VEGF-B exclusively, mediated by the receptor VEGFR-1 through receptor tyrosine phosphorylation, which subsequently leads to activation of the major downstream signaling pathways. In accordance with previous findings of our group in colorectal carcinomas, lack of total VEGFR-1 in colonic tumor cells was significantly associated with lymphogenous CC metastasis [40]. In the current study, absence of tumoral pVEGFR-1^Tyr1048^ and pVEGFR-1^Tyr1213^ expression as well as VEGFR-1/pVEGFR-1 co-expression in the tumor center was observed in cases with distant metastasis. Moreover, in tumor budding regions lack of VEGFR-1/pVEGFR-1 co-expression was associated with haematogenous and lymphogenous metastasis. These data indicate that VEGFR-1 autophosphorylation at Tyr1048 or Tyr1213 is acting as a negative regulatory mechanism for processes facilitating CC metastasis. Because of the identical directional association of PlGF overexpression with the metastatic status and the close correlation between PlGF and VEGFR-1 expression, we assume that this ligand could be a potential link of an autocrine loop having a protective effect in colon carcinoma cells themselves. To the best of our knowledge other detailed data about the expression pattern of pVEGFR-1 *in situ* for CC and generally for malignant tumors are not yet to be found in the literature. Our results concerning the correlation between VEGFR-1 downregulation and CC metastasis are in accordance with those of Hanrahan et al., who found significantly increased VEGFR-1 levels in CC without metastasis in comparison with nodal-positive cases [17]. Furthermore, Garouniatis et al. concluded from their studies that loss of VEGFR-1 predict distant metastasis in CC [41]. In contrast, Wei et al. reported that PlGF expression correlates with VEGFR-1 expression, and high m-RNA levels of both are associated with CC progression [33]. In the light of these contrasting findings a detailed analysis with regard to the different cellular components expressing this receptor could be insightful. VEGFR-1, pVEGFR-1^Tyr1048^ and pVEGFR-1^Tyr1213^ were also found in blood vessels of all vascular segments, but only the macro vessels displayed significant differences between the comparative tumor fractions. Thus, significantly more non-metastatic CC revealed VEGFR-1/pVEGFR-1 co-expression in small vessels in the tumor center or along the invasive front. This suggests that VEGFR-1 activation of the tumor-associated branched vascular network protects against CC metastasis. Whether this is related to a regulation of tumoral hypoxic conditions cannot be assessed at this time. Vascular pVEGFR-1^Tyr1333^ expression seems to play a negligible role. Interestingly, all cases with tumor-cell associated positivity showed a nuclear expression of pVEGFR-1^Tyr1333^. It is known that receptor tyrosine kinases are also transported to the nucleus, where they may directly impact nuclear signaling [42]. Ancillary molecular studies are necessary to verify the status of the phosphorylated receptor location in the cellular compartments.

Our study had several limitations, including a relatively small number of investigated cases and the exclusive use of immunohistochemistry as detection method, although the detection of phosphorylated moieties does yield some functional data. Thus, up to now our study represents the first cohort to be investigated in such detail and the present data enable an initial assessment of the role of the VEGFR-1 pathway for colon cancer metastasis. In this respect a major advantage of the immunohistochemical detection method is the precise identification of both tumoral and non-tumoral histological structures and their topological distribution.

## Conclusion

Collectively, our study indicates that the total and phosphorylated VEGFR-1 and its ligands participate in vascular, tumor cell-mediated and immuno-inflammatory processes in a biomolecule-specific and tumor zone-specific manner to prevent or to promote metastasis in CC. Figure 5 summarizes these complex mechanisms of the VEGFR-1 activating pathway in CC tissue. PlGF-mediated autocrine VEGFR-1 activation in tumor cells and paracrine receptor activation in small vessels within the tumor and along the invasive margin seem to have an inhibitory effect on metastasis. PlGF/VEGFR-1 co-expression in extratumoral small vessels and receptor expression in inflammatory cells at the invasive front appear to be associated with a non-metastatic phenotype. VEGF-B expression in tumor cells and extratumoral macrovasculature is strongly associated with metastasis. In contrast, VEGF-B-expressing small vessels in the tumor center could possibly reduce the metastatic potential of CC.
